# Surgery

**Published:** 1905-09-09

**Authors:** 


### PROGRESS IN MEDICINE AND SURGERY.

### SURGERY.

(*Continued from page 395.*)

**On Bone Grafting**—Huntingdon [4] had a patient, a boy of seven, under his care who, by osteomyelitis of the left tibia, lost practically the whole shaft of the bone. After the diseased bone had been removed the periosteum was carefully preserved, but nine months later a gap of more than five inches still remained between the two ends of the bone, the limb being loose and flail-like. The fibula was sawn through at the level of the lower end of the upper tibial fragment, and the divided end of the fibula firmly fixed into a cup-shaped depression in the head of the tibia. Slow union occurred, but the weight of the body could not be properly borne on the limb. Therefore, ten months later, the lower end of the fibula was sawn through and fixed into the distal fragment of tibia. As a result of this the boy had a sound and useful limb.

**Wounds of the Thoracic Duct.**—P. Lesène [5] discusses twenty-two recorded cases of this injury, dividing them into those where the accident is recognised at the time of operation and those in which it is only evident afterwards by an escape of chyle through the wound. If recognised at the time when it is cut, it is only necessary to tie the peripheral end of the duct. When the condition is manifested later, symptoms arise of intense thirst, emaciation, weakness, and sometimes oliguria. Graduated compression over the wound is generally sufficient to cure the condition. Only one case has proved fatal. In one case the partially severed duct was successfully sutured.

**On the Treatment of Cavities in Bones and Joints.**—For five years Moorhof, of Vienna, has been trying a new method, and has treated altogether 79 cases of osteomyelitis, 108 cases of tuberculous disease, two fillings of the antrum of Highmore, and four of dental cysts. Seymour Jones [6] describes the technique of the process and the operative details of various cases. The plugging substance consists of 60 parts iodoform and 40 parts each of spermaceti and oil of sesame, which is solid at ordinary temperatures and liquefies at 112° F. The part containing the diseased bone is emptied of blood by elevation, elastic bandaging and tourniquet, the cavity is thoroughly scraped out, so that all infected material is removed, washed with a dilute solution of formalin and dried with a stream of hot air. The iodoform-wax is then poured in hot and allowed to solidify and the skin sewn together over it.

It acts as a scaffolding for the building of granulation tissue, and finally bone, and the process can be followed by the *x*-rays. The wax gives a dark shadow on the skiagram. In about eight weeks the edges of this begin to get lighter, and a mass the size of a hen's egg completely disappears and is replaced by bone within one year. Besides the cavities left in bones by necrosis and abscess, tuberculous joints in all stages are considered suitable for this treatment, and, with the exception of the hip, all the joints of the limbs have been successfully plugged after extensive arthrectomies had been performed. Of nearly 200 cases in which the method has been adopted it has failed in none.

**On Some New Antiseptics.**—Iodic acid and the iodic salts when applied to wounds liberate iodine in a very active state, in which it acts as a powerful germicide. Mackie[7] speaks very highly of calcium iodate used as a dusting powder, as a 10 per cent. ointment, and as a 3 per cent. gauze. Septic bone injuries and suppurating wounds granulated rapidly under its use. As a douche for septic conditions of the vagina, bladder, otorrhœa, and pyorrhœa alveolaris, it rapidly promoted a cure. As an injection into tuberculous joints it seems to be more active than iodoform. Iodic acid itself seems to be an even stronger antiseptic, so that, when added to urine in the proportion of 1 in 2,500, which was then exposed to the air, it prevented the development of any decomposition for an indefinite period. Sub-iodate of bismuth as a dusting powder has been used very successfully in the treatment of lupus after scraping, and mercuric iodate in an ointment of 20 grains to the ounce for obstinate sycosis. A more familiar instance of the activity of an element when in the "nascent state" is that of oxygen when liberated from the peroxides, and although hydrogen peroxide has been long used for this reason as a powerful disinfectant, the sudden and copious liberation of oxygen is a drawback. Schuh[8] draws attention to the much greater convenience of dioxide of zinc in this respect. It can be used as a dusting powder or a 10 per cent. ointment, and in either case a constant stream of active oxygen is slowly given off when in contact with wound surfaces. In all kinds of chronic ulcers and foul suppurating wounds it is therefore of the greatest value.

**On the Reduction and Fixation of Fractures of the Jaws.**—The difficulty of this subject has led to the introduction of many and various devices. Lederer[9] takes a model of both upper and lower jaws, and from these makes a splint of tin or rubber which separates the jaws for about ¾-inch. The splint is introduced under an anæsthetic, the fracture reduced by forcing the fractured portion into the splint, and the jaws are bandaged together by roller bandages. The opening of the jaws permits of fluid feeding. Matas[10] has constructed a splint in three portions—one (made in three sizes to fit different aged patients) of malleable tin lies over the lower teeth, embracing the whole series, any gaps being filled by the application of modelling composition to the interior of the trough dental splint; the second is a chin piece; and the third a clamp made of a jointed steel elbow. The ends of the elbowed clamp fit into slots in the dental and chin splints, and the arms of the clamp are then screwed together by a thumbscrew. This arrangement does not interfere with the opening of the mouth, feeding, or oral irrigation.

**Dislocation of the Patella with Horizontal Displacement.**—Cheeseman[11] relates the following case. A boy of 13 had been thrown from a moving railway train and fell on his left knee. The patella could be felt tilted up with the lower edge pointing forwards, and so firmly fixed that no external manipulations could replace it. An open operation was performed, and the quadriceps insertion into the patella found to be completely severed. The same force which tore the tendon must have driven the upper edge of the bone into the angle between the femoral condyles and the tibia. It was levered out of this position by a director pushed through the ligamentum patellæ, and the quadriceps tendon sewn to the patella by catgut. Recovery with good movement was complete.

[4] Annals of Surgery, Feb. 1905. [5] Edin. Med. Jour., Feb. 1905. [6] Lancet. Jan. 21, 1905. [7] Ibid. [8] Nova Medica, Jan. 1905. [9] Med. Chron., Feb. 1905. [10] Annals of Surgery, Jan. 1905 [11] Ibid.

